# The River Ambulance Service

**Published:** 1914-04-04

**Authors:** 


### The River Ambulance Service.

We are in a position to add more exact particulars to the reference which was recently made in these columns to this interesting branch of the work of the Metropolitan Asylums Board.

The River Ambulance Service of the Metropolitan Asylums Board was originated in 1884 for the purpose of conveying smallpox patients from riverside wharves in London, used as receiving and diagnosing stations, to the smallpox hospital ships lying in Long Reach, near Dartford. It now consists of a fleet of five steamers, four of large size, and capable of carrying a number of patients varying from about twenty to nearly seventy, according to the size of the boat, and one, smaller, for four patients.

The smallpox hospital ships were superseded in 1904 by the Long Reach and Joyce Green Hospitals, and these, being adjacent to the river, continue to be served by the River Ambulance Service. The former of these hospitals is still reserved for smallpox patients, but Joyce Green Hospital is now in use for fever when not required for smallpox purposes, and the River Service has been used during the last few months for the transport from the London hospitals of the Board to Joyce Green Hospital of convalescent and mild acute cases of scarlet fever and of convalescent diphtheria and whooping-cough patients, and, to a less extent, for the conveyance of cases of scarlet fever and of whooping-cough admitted direct from their homes without first passing through one of the acute fever hospitals. It was also used largely in connection with the discharge of patients from the hospitals at Dartford.

The steamers are specially fitted for these purposes, being, in fact, miniature hospitals, and, realising the ease and comfort to the patients which they afford, the Managers will, in all probability, make extended use in the future of this means of transport between London and the hospitals at Dartford.

